# Cumulative genetic score of *KIAA0319* affects reading ability in Chinese children: moderation by parental education and mediation by rapid automatized naming

**DOI:** 10.1186/s12993-023-00212-z

**Published:** 2023-06-01

**Authors:** Jingjing Zhao, Qing Yang, Chen Cheng, Zhengjun Wang

**Affiliations:** School of Psychology, Shaanxi Normal University and Shaanxi Provincial Key Research Center of Child Mental and Behavioral Health, Yanta District, 199 South Chang’an Road, Xi’an, 710062 China

**Keywords:** *KIAA0319*, Reading ability, Rapid automatized naming, Differential susceptibility model, Gene-environment interaction

## Abstract

*KIAA0319*, a well-studied candidate gene, has been shown to be associated with reading ability and developmental dyslexia. In the present study, we investigated whether *KIAA0319* affects reading ability by interacting with the parental education level and whether rapid automatized naming (RAN), phonological awareness and morphological awareness mediate the relationship between *KIAA0319* and reading ability. A total of 2284 Chinese children from primary school grades 3 and 6 participated in this study. Chinese character reading accuracy and word reading fluency were used as measures of reading abilities. The cumulative genetic risk score (CGS) of 13 SNPs in *KIAA0319* was calculated. Results revealed interaction effect between CGS of *KIAA0319* and parental education level on reading fluency. The interaction effect suggested that individuals with a low CGS of *KIAA0319* were better at reading fluency in a positive environment (higher parental educational level) than individuals with a high CGS. Moreover, the interaction effect coincided with the differential susceptibility model. The results of the multiple mediator model revealed that RAN mediates the impact of the genetic cumulative effect of *KIAA0319* on reading abilities. These findings provide evidence that *KIAA0319* is a risk vulnerability gene that interacts with environmental factor to impact reading abilities and demonstrate the reliability of RAN as an endophenotype between genes and reading associations.

**Supplementary Information:**

The online version contains supplementary material available at 10.1186/s12993-023-00212-z.

## Introduction

Reading ability is a complex behavioral characteristic that mainly includes reading accuracy and reading fluency. The acquisition of reading skills are associated with series of reading-related linguistic processes, such as phonological awareness, orthographic awareness, morpheme awareness and rapid automatized naming [1]. It is known that genetic variation accounts for 20–80% of the total variation in reading skills, and the genetic variations discovered thus far only explain the “tip of the iceberg” of estimated heritability [1, 2].

Among the susceptibility genes of reading dis/ability identified to date, *KIAA0319* is an important candidate gene. *KIAA0319* affects neuronal migration, neurite outgrowth, cortical morphogenesis, and ciliary structure and function [3–6]. Schmitz et al. analyzed DNA methylation in the *KIAA0319* promoter region to investigate whether epigenetic markers of language lateralization could be identified in nonneuronal tissue [7]. These data provide a framework to interpret the effects of the dyslexia-associated genetic variants that reside in *KIAA0319* noncoding regulatory regions [8].

Franks et al. used quantitative trait loci (QTL) analysis and found that single nucleotide polymorphisms (SNPs) in *KIAA0319* were associated with word reading, phonological awareness, and orthographic rules [9]. Moreover, rs2143340 in *KIAA0319* was replicated in the Avon Longitudinal Study of Parents and Children (ALSPAC) and found to be associated with poor performance of reading and spelling [10]. In subsequent studies, Scerri et al. found that rs2143340 was significantly correlated with word reading accuracy in their quantitative analysis of reading phenotypes [11]. Quantitative analysis of samples from Dutch children with developmental dyslexia found that rs761100 and rs2038137 in *KIAA0319* were related to digit rapid automatized naming, and that rs6935076 in *KIAA0319* was related to word reading fluency [12]. However, there were also studies reporting no significant relationship between *KIAA0319* and reading ability [13, 14].

These inconsistencies can be explained in part by heterogeneity between studies, possibly due to the different criteria for phenotypic assessment, age, sample size, population genetic background, and environmental factors. On the one hand, environmental factors can influence the probability of gene expression in behavior [15].

Studies have shown gene‒environment interactions on several environmental factors, e.g., the home literacy environment (HLE), socioeconomic status (SES), the prenatal education, and computer game interventions [14, 16–18]. For example, the presence of an interaction between maternal stress during pregnancy and the rs12193738 polymorphism in *KIAA0319* was shown to affect reading ability at 16 years of age [16]. However, one polymorphism cannot represent the variation in gene function, and many variants with small effects cannot be detected. The cumulative genetic score (CGS) can be used to investigate the influence of multiple genetic polymorphisms [19–22]. The CGS was combined into a single score by assigning points (0, 1 or 2) according to the number of sensitive alleles [23]. Using the CGS can increase statistical power and model more variants on a gene. Therefore, the first aim of the present study was to use the CGS approach to explore whether the CGS of *KIAA0319* impacts reading ability by interacting with the environmental factor of parental education level.

Nevertheless, three gene‒environment models (G × E) exist at present. The diathesis-stress model emphasizes that individuals with disease risk alleles or vulnerability genotypes have higher negative environmental sensitivity [24, 25]. The vantage sensitivity model aims to describe the different responses of individuals in positive environments [25]. According to the differential susceptibility model, the same genetic traits or genotypes also have the effect of making individuals better (for better) or worse (for worse) under environmental factors [26]. To explore whether *KIAA0319* should be considered a vulnerable or plastic gene [27], the second aim of the present study was to conduct interaction and competing model analyses to examine which gene-environment model of the interaction between *KIAA0319* and parental education follows.

On the other hand, when studying the role of candidate genes, endophenotypes (EPs) can also reduce the effect of heterogeneity [15]. Endophenotypes are more useful than macroscopic behavior indicators and serve as mediating variables for understanding complex pathways [28, 29]. EPs reflect neurophysiological, biochemical, endocrinological, cognitive or neuropsychological processes that are associated with traits or diseases and may reflect specific genes that are associated with behavioral phenotypes [30–32].

Reading-related linguistic skills might be important EPs between candidate genes and reading abilities. Rapid automatized naming (RAN), a well-studied reading-related skill, has been widely used in studies of reading acquisition and has been found to be reliably related to reading achievement [33, 34]. Several studies have identified RAN to be independent of reading and reading-related cognitive processes, such as orthographic processing, phonological awareness, and short-term memory [35–37]. Moreover, RAN has been found to be heritable. Genome-wide association studies reported the effects of SNPs on RAN, and classical twin studies found heritability estimates ranging from 0.56 to 0.70 for RAN [38–42]. The latest results explored RAN as an endophenotype that mediates the association between genes and reading ability [14, 43]. Therefore, RAN was investigated as a mediator between *KIAA0319* and reading ability in the present study.

Phonological awareness and morphological awareness have also found to contribute to the development of reading ability and significantly predict word recognition and reading speed [44–47]. However, as reading-related linguistic skills, no studies have explored the possibility of phonological awareness and morphological awareness as endophenotypes. The heritability estimates of phonological awareness and morphological awareness were 0.19–0.83 [40, 48, 49] and 0.44–0.55 [50], respectively. Candidate gene studies have also discussed the association of *KIAA0319* with phonological and morphological awareness [51, 52]. Therefore, this study also aimed to investigate the mediating effects of phonological awareness and morphological awareness as intermediate phenotypes.

**Fig. 1:**
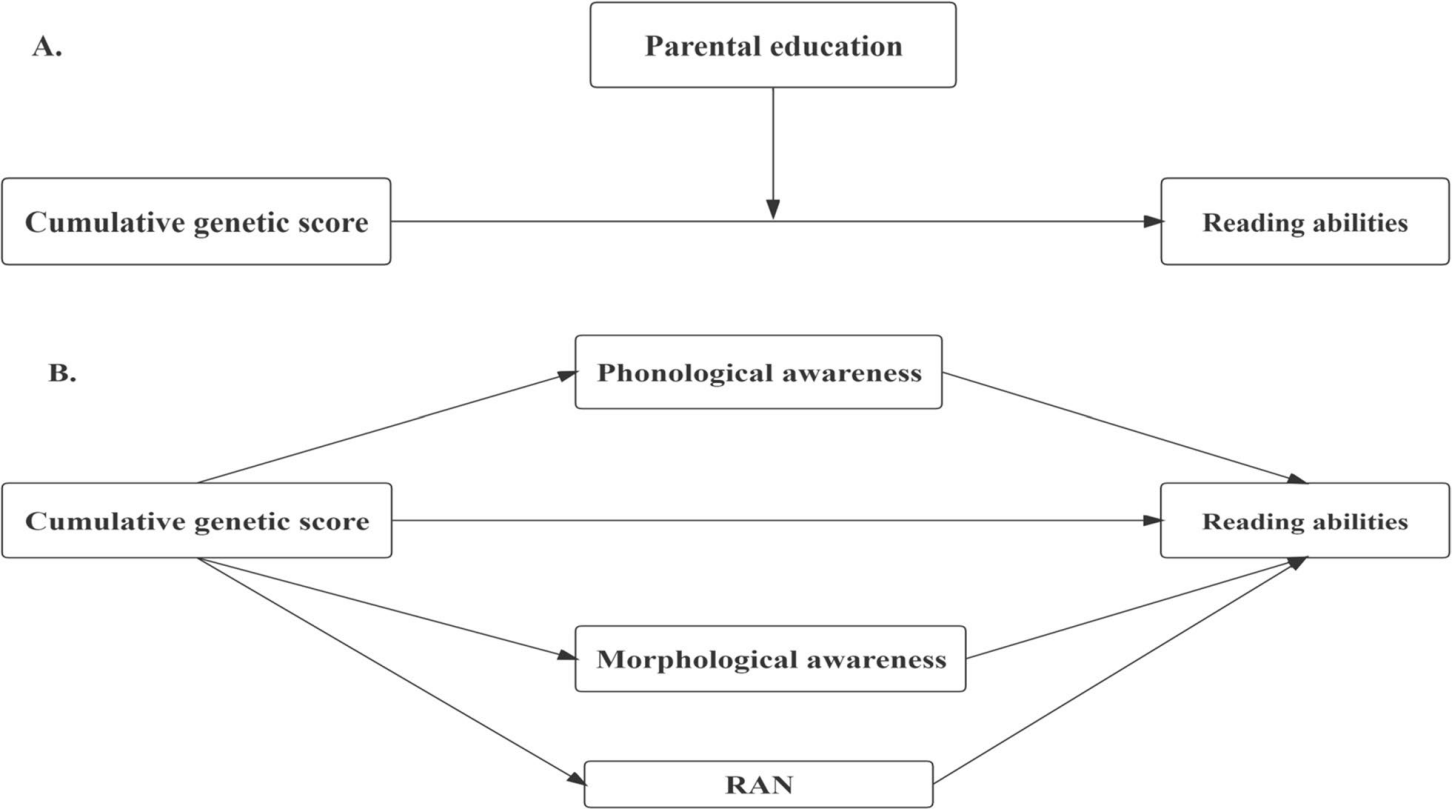
The plot of the moderation effect of environment in genetic influence on reading-related phenotypes (**A**); the multiple mediation model plot of cognitive skills in gene-phenotype association (**B**)

**Fig. 2:**
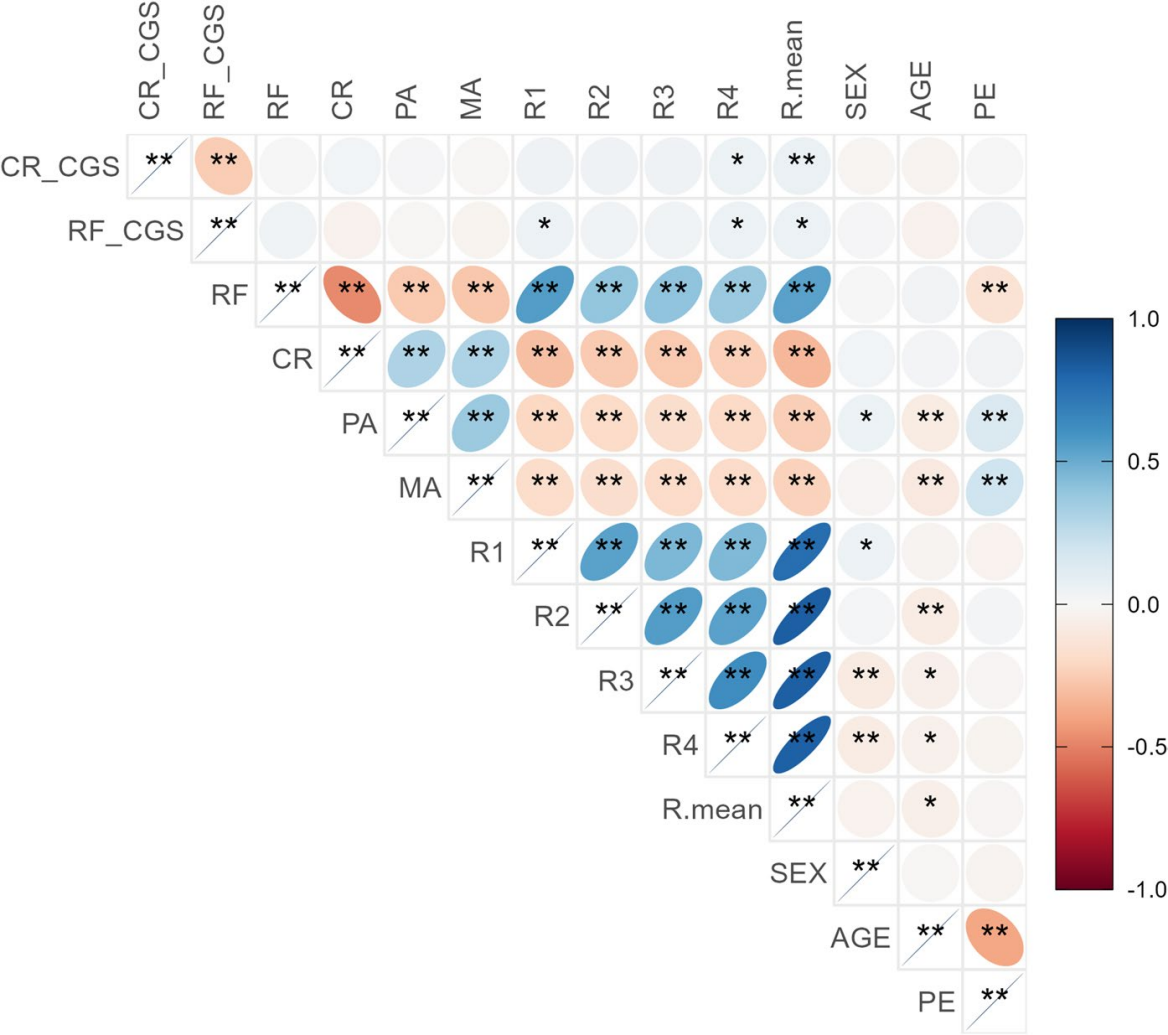
The heat map of Pearson correlation coefficient and p-value between CGS and behavioral phenotypic variables. *CR_CGS:* the cumulative genetic socre of *KIAA0319* on character recognition, *RF_CGS:* the cumulative genetic socre of *KIAA0319* on reading fluency, *RF:* reading fluency, *CR:* character recognition, *PA:* phonological awareness, *MA:* morphological awareness, *R1:* digit rapid automatized naming, *R2:* dice rapid automatized naming, *R3:* picture rapid automatized naming, *R4:* color rapid automatized naming, *R.mean:* the mean Z-score of four RANs, *PE:* parental education. *p < 0.05, **p < 0.01

In sum, we aimed to investigate 1) the effect of the *KIAA0319* interaction with the environment on reading abilities (Fig. 1A); 2) which gene‒environmental interaction model better fits the G × E effect; and 3) whether genes affect reading abilities through endophenotypes of rapid automatized naming, phonological awareness, and morphological awareness (Fig. 1B).

## Materials and methods

### Participants

A total of 2284 participants (primary school students from Shaanxi, Gansu and Inner Mongolia) were recruited. All participants were normal school-age students from grade 3 to grade 6 without any history of mental illness. In this study, saliva samples were collected (gene samples were extracted). Participants completed two reading tests, including Chinese character recognition task (N = 2270) and Chinese word reading fluency task (N = 2270), six reading-related linguistic tasks, including four rapid automatized naming tasks (digit, picture, color, and dice) and phonological awareness and morphonological awareness tasks (N = from 1968 to 2244).

**Table 1:** The interaction between the cumulative genetic scores of KIAA0319 and parental education on reading fluency

Parameter	Gene(G) and environment(E) main effects: Model 1	Main effects and G × E interaction: Model 2
*B*_*0*_	253.53 (10.34)	270.10 (12.70)
*B*_*1*_	−5.27 (0.73)	−10.41 (2.40)
*B*_*2*_	0.51 (0.28)	−1.20 (0.81)
*B*_*3*_	–	0.53 (0.24)
*B*_*4*_	−0.83 (0.07)	−0.83 (0.07)
*B*_*5*_	−2.06 (1.60)	−2.12 (1.60)
*R*^*2*^	0.085	0.088
*F*	36.69	30.43
*df*	4, 1583	5, 1582
*p*	< 0.0001	< .0001
*F* vs. 1	–	4.991
*df*	–	1,1581
*p*	–	0.0256

### Genotyping and SNP selection

An Illumina Asian Screening Array (ASA, 700 K–750 K) chip was used for genotyping the obtained DNA samples using the Beijing Compass biotechnology formula. PLINK was used to screen the following genotypes for standard quality control (Aderson et al., 2010; Chang, 2015): a single sample SNP detection rate higher than 0.9 (sample call rate > 0.90); single SNP detection rates (SNP call rate > 0.95); Hardy–Weinberg equilibrium coefficients (*p* < 10^–5^); minor allele frequencies (MAF > 0.01); and those with first degree of kinship was removed (PI_HAT > 0.50). MACH 4.0 software was used to carry out full genome data (imputation) analysis based on the Asian population data in the Genome Asia Pilot (GAsp) project, and the filled data were consistent with the previous quality control standards. Thirteen SNPs were extracted by PLINK v1.90.

### Phenotypes

*Reading fluency (RF).* Wordlist reading task [53, 54] was used to measure each child’s reading fluency. In this task, children were asked to name a list of 180 two-character words as rapidly and accurately as possible. All these words were from primary school text books and have been learned before grade 3, such as “我们(we)” and “太阳(sun)”. Since words included in this task were all simple, this task was administrated to test children’s reading fluency. The total time for naming the whole word list was recorded as measurement of reading fluency.

*Character recognition (CR):* Chinese character recognition test was used to measure each child’s reading accuracy [53, 54]. The test consisted of 150 single Chinese characters selected from China’s Elementary School Textbooks (1996), with a reliability of 0.95 [53]. Each child was individually tested and required to read aloud each character at a time.

*Rapid automatized naming tasks.* The rapid automatized naming tasks include four tasks: rapid automatized naming of digits, pictures, colors, and dices [54]. Four series of 40 items (digits, pictures, dices, and colors) were presented to each child, with each type of items on a separate sheet of paper. The digits (2, 4, 6, 7, and 9) were used as stimuli of rapid automatized digit naming task. The pictures (dog, flower, book, shoe, and window) were used as stimuli of rapid automatized picture naming task. Pictures of dices (one, two, three, four, and five) were used as stimuli of rapid automatized dice naming task. The colors (red, yellow, black, green, and blue) were used as stimuli of rapid automatized color naming task. Each sheet includes eight rows with five items in a row. Children were required to name each type of items as rapidly as possible. Each child named twice for each sheet. The measurement of rapid automatized naming was the average naming times for the two times of each type of items. The test–retest reliabilities of rapid automatized digit, picture, dice, and color naming tasks were 0.87, 0.82, 0.74, and 0.74, respectively.

*Phonological awareness task.* In this task, a child is verbally presented with a one-syllable word. The child’s task is to remove a given phoneme from the syllable in the word and speak out the rest of the syllable. The task consists of 16 items: initial phoneme deletion items (e.g., /mei4/ (sister) without /m/), middle phoneme deletion items (e.g., /tuan4/ (group) without /u/), and final phoneme deletion items (e.g., /guan1/ (close) without /n/). This task has been widely used in language studies of Chinese children [46, 55, 56]. The reliability (Chronbach’s alpha) of the test was 0.90 [54].

*Morphological awareness task*. Children are asked to identify one of the morphemes among two-morpheme words and to create two new words with the target morpheme [55, 57]. One of the morphemes in a word has the same meaning as the target morpheme; conversely, one of the morphemes in the other word has a different meaning. Presented with the word /bei1 bao1/ (which means backpack), children are asked to produce two new words containing /bao1/. In one word, /bao 1/ has the same meaning as /bei1 bao1/, such as /shu1 bao1/ (which means bag). And in the other word, /bao 1/ is different from that of /bei1 bao1/, such as /bao1 zi1/ (which means steamed stuffed bun). The Cronbach’s alpha of this questionnaire was 0.80 [57].

### Parental Education (PE) levels

A total of 1620 participants in this study had information about their parents' educational levels, with 1 representing the lowest educational level and 8 representing the highest educational level: 1 = primary school education, 2 = junior high school education, 3 = senior high school education, 4 = junior college education, 5 = undergraduate degree, 6 = master’s degree, 7 = doctoral degree, and 8 = postdoc. The average score of mother’s and father’s educational levels was used as a child’s parental educational level. Finally, 1,588 children had data on both character recognition and parental educational level, and 1,589 children had data on both reading fluency and parental educational level.

### Data analysis

#### SNP coding and cumulative genetic scores (CGSs)

The SNPs on *KIAA0319* were reported in our recent GWAS of dyslexia and were replicated in reading fluency in the Chinese sample [58, 80]. The sample of the current study and the Chinese sample in Doust et al. were from the same cohort [58]. We therefore adopted the β values for the phenotype of reading fluency and character recognition from our Chinese sample in this study [80]. The cumulative genetic score (CGS) of the *KIAA0319* gene was calculated by combining risk alleles of the 13 SNPs. Coding was based on the first allele and beta value. When the value of β was positive, the homozygous with the first allele was 2 and the heterozygous was 1, 0 was the homozygous for the minor allele. When the beta value was negative, it was opposite to the encoding genotype.

#### Gene by Enviornment Interaction analysis

Stratified regression analysis was performed to explore the interactions of 13 SNPs and the CGS with the parental education level. The standard multiple regression equation is as follows:

1 $$Y = B0 + B1X1 + B2X2 + B3(X1 \times X2) + B4 \cdot Age + B5 \cdot Sex + E$$

where Y is the dependent variable (i.e., reading fluency); *X*_*1*_ is the environmental variable (PE); *X*_2_ is the genetic variable, *X*_*1*_ × *X*_*2*_ is the product term of the gene‒environment interaction; *B*_*1*_ and *B*_*2*_ are the regression slopes of the main effects of environment *X*_*1*_ and gene *X*_*2*_, respectively; *B*_*3*_ is the regression coefficient of their interaction term; *B*_*0*_ is the intercept; and *E* is the random error.

#### Reparameterized regression model tests

The reparametric equation [59] is as follows:

2 $$\begin{gathered} \hfill \\ Y = B0 + B1(X1 - C) + B2((X1 - C) \times X2) + B3 \cdot Age + B4 \cdot Sex + E \hfill \\ \end{gathered}$$

Equation 2 is a 5-parameter equation (i.e., B0, B1, B2, B3, B4, C). C is the intersection of the predicted values of the environmental variables of the two groups; if the crossover of C and its confidence interval (CI) is within the range of values in the environment, the interaction is disordinal, reflecting the differential susceptibility model, and if it falls outside the range, the interaction is ordinal and conforms to the diathesis-stress model [59].

In this study, B1 is the slope of PE, and B2 is the slope of the interaction term. Point C is not fixed. If Point C is within the range of the parental education level, the interaction of G × E conforms to the differential susceptibility model. If Point C is fixed, a crossover point that falls at the maximum value of the environment variable is added (C = Max (PE)). At this point, the interaction of G × E is orderly and conforms to the diathesis-stress model. ANOVA, the Akaike information criterion (AIC) and the Bayesian information criterion (BIC) were used to evaluate the two models. For the AIC and BIC, the lower the value is, the higher the efficiency of the model.

#### Mediation analysis

The series of structural equation modeling (SEM) analyses were conducted by using Mplus 17.0 to explore whether rapid automatized naming, phonological awareness, and morphological awareness mediate the effect of cumulative genetic scores on reading fluency and reading accuracy after controlling for age and sex. All the phenotypes and endophenotypes were transformed into Z score according to separately each grade [54]. All the predictor measures were allowed to be related to each other. Indirect effects were tested using the 5000 bootstrap technique [60], and confidence intervals (95% CIs) that did not contain zero indicated significant indirect effects [61]. We reported a variety of indices to reflect the model fit [62].

## Results

### Correlation analysis

The 13 SNPs conformed to Hardy–Weinberg equilibrium (Additional file 1: Table S1). Figure 2 presents the correlation among variables for the total sample. Due to correlations between behavioral phenotypic variables, we adopted FDR correction (Benjaminiand Hochberg correction) for multiple corrections to reduce the type I error (Additional file 1: Table S2). The CGS of reading fluency (RF_CGS) was marginally correlated with reading fluency (r_*p*_ = 0.040, *p* = 0.06) and significantly correlated with digit rapid automatized naming (r_*p*_ = 0.052, *p* = 0.02). The CGS of character recognition (CR_CGS) were marginally significantly correlated with color rapid automatized naming (r_*p*_ = 0.053, *p* = 0.02). For the mean Z-score of the four RAN tasks, it was significantly correlated with CR_CGS (r_*p*_ = 0.061, *p* = 0.007), and significantly correlated with RF_CGS (r_*p*_ = 0.055, *p* = 0.02). Reading fluency and all of the rapid automatized naming tasks were significantly correlated. Moreover, there was no correlation between PE and genetic variables.

### Standard exploratory analysis

**Fig. 3:**
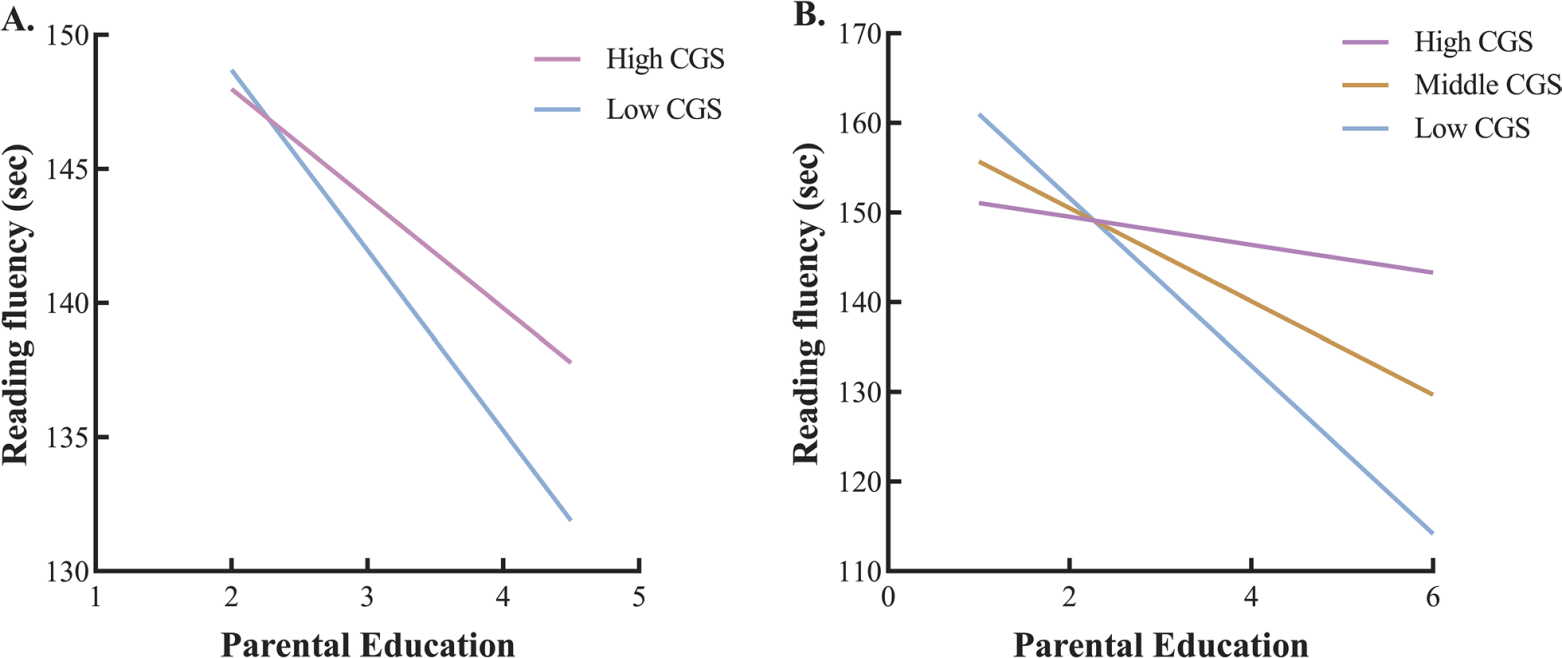
Simple slope analysis of reading fluency in the Low and High CGS subgroups (**A**); the plot for the results of the interaction between the CGS and PE level to predict reading fluency in the differential susceptibility model (**B**)

Standard regression equations were used to test the G × E effect of a single SNP, CR_CGS (Additional file 1: Table S3 and Table S4) and RF_CGS (Table 1). In reading fluency and character recognition, no single SNP reached significance in the interaction terms after Bonferroni correction (*adjusted-p*: *0.05/13* = *0.0038*). PE level was significant in all regression main effects. In Model 1 (Table 1), before adding the G × E interaction term, the main effect of the PE level was significant (*B*_*1*_ = *-5.27*,* p* < 0.001), and the predicting effect of RF_CGS for reading fluency was not (*B*_*2*_ = *0.51, p* = *0.07*). In Model 2, the G × E effect was significant (*B*_*3*_ = *0.53, p* = *0.026, R*^*2*^ = *0.088)*. Simple slope analysis showed (Fig. 3A) that with the increase in the PE level, the time of reading fluency of the low RF_CGS group was significantly faster than that of the high RF_CGS group. However, the interaction between CR_CGS and the PE level did not predict character recognition.

### Competing test selection

Reparameterized Eq. 2 was used to verify the interaction between the *KIAA0319* gene and parental education level. Table 2 shows that Model 3 (i.e., the differential susceptibility model) had the best fitting effect on reading fluency (Fig. 3B). Compared with Model 4, Model 3 had an estimated parameter added, and the interpretation rate of R^2^ increased significantly (△R^2^ = 0.004, *p* = 0.006), so Model 4 was rejected. Furthermore, by comparing Model 3 and Model 4, the AIC and BIC of Model 4 were both larger than those of Model 3, and Model 3b was rejected. Overall, Model 3 had better fitting performance. In the G × E effect, RF_CGS and the parental education (PE) level fit the differential susceptibility model.

**Table 2:** Re-parameterized regression analysis

Linear × Linear interaction		
Parameter	Differential susceptibility Model 3	Diathesis-Stress Model 4
*B*_*0*_	246.63 (9.70)	227.35 (8.24)
*B*_*1*_	−10.40 (2.40)	−4.57 (1.16)
*B*_*2*_	0.52 (0.24)	−0.07 (0.09)
*C*	2.27 (0.68)	6.00 (–)^a^
*B*_*3*_	−0.83 (0.07)	-0.83 (0.07)
*B*_*4*_	−2.13 (1.60)	−2.05 (1.60)
*R*^*2*^	0.0875	0.0831
*F*	31.85	31.92
*df*	5,1581	4, 1582
*p*	< 0.0001	< 0.0001
*F* vs. 2	7.65	–
*df*	1,1581	–
*p*	0.006	–
AIC	15497.49	15503.15
BIC	15535.08	15535.37

Tabled values are parameter estimates, with their standard errors in parentheses

*F* vs 2, stands for an* F* test of the difference in R^2^ for Model 3 versus Model 4, respectively

*AIC* Akaike information criterion, *BIC* Bayesian information criterion

^a^Parameter fixed at maximum deviation; SE is not applicable, so is listed as (–)

### Test of the mediation model

**Fig. 4:**
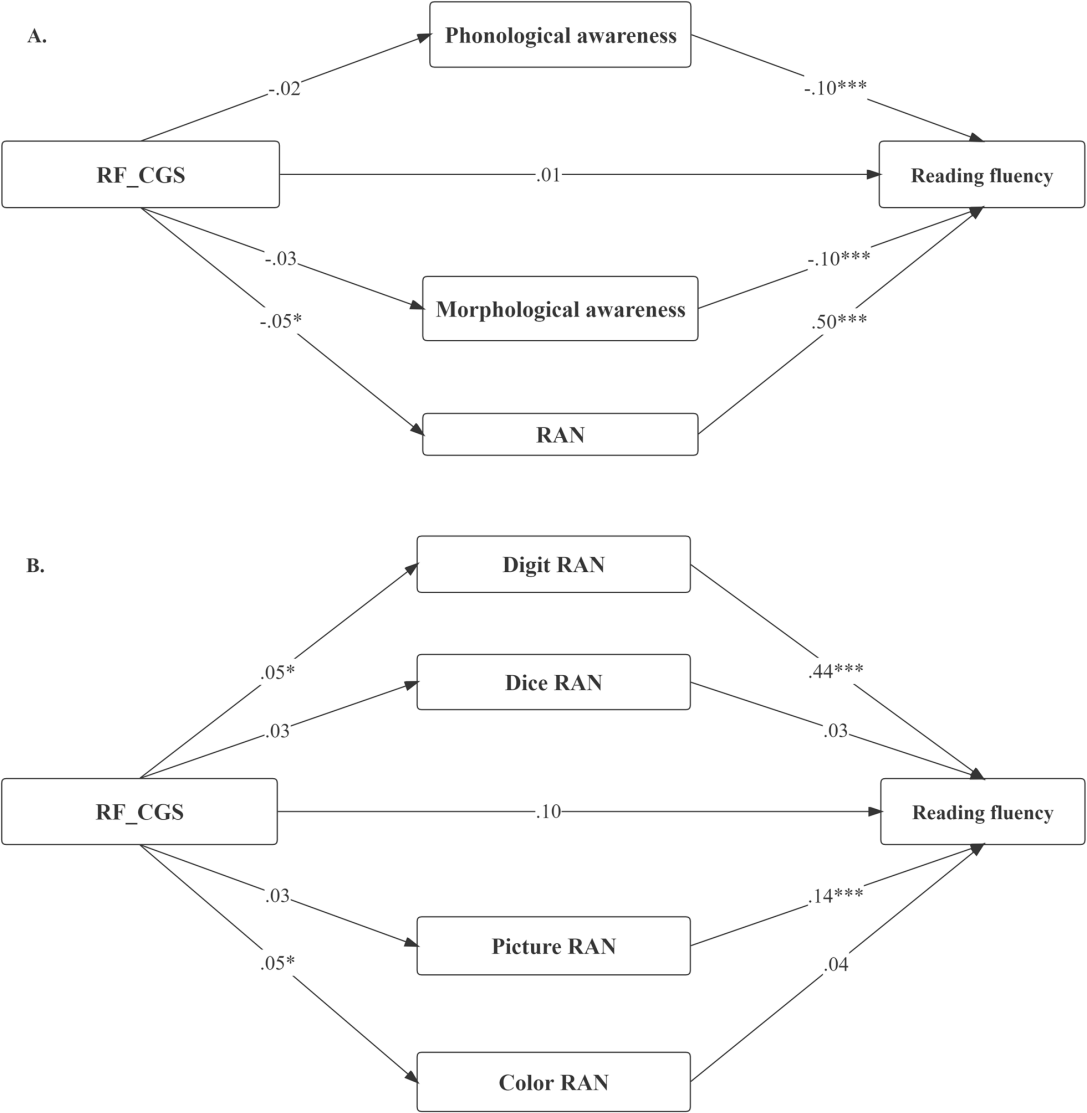
Significant specific indirect of the RAN effect from RF_CGS to reading fluency after controlling for sex and age (standardized estimates of the path coefficients are depicted in Model 5) (**A**); Significant specific indirect of the RAN effect from RF_CGS to reading fluency after controlling for sex and age (standardized estimates of the path coefficients are depicted in Model 6) (**B**).The model was adjusted because of the correlations between individual RAN (some path coefficients have been omitted for brevity). RAN: the mean Z-score of four RAN tasks

A series of structural equation modeling (SEM) analyses were conducted to explore whether rapid automatized naming, phonological awareness, and morphological awareness mediated the effect of the *KIAA0319* gene on reading fluency after controlling for sex and age (Fig. 4). The indices of the Model 5 in Table 3 provided a good fit to the data: the CFI (comparative fit index) = 0.997 > 0.90; the TLI (Tucker‒Lewis index) = 0.981 > 0.90; the RMSEA (root mean square error of approximation) = 0.024 < 0.80; and χ^2^/*df* = 2.35 < 3. Using 5000 bootstrap analyses and 95% CIs, we found that the significant mediation of RAN (average z score of the four RAN tasks) in reading fluency (*p* = 0.034), and the 95% CI [0.001, 0.017], did not include 0 (Model 5 in Table 4, Fig. 4A) and total indirect effect was significant (*p* = 0.021, *95% CI* [0.001, 0.019]).

**Table 3:** Fitting index of KIAA0319_CGS on reading abilites of mediation model

	χ^2^	df	χ^2^/df	CFI	TLI	RMSEA
Model 5	7.05	3	2.35	0.997	0.981	0.024
Model 6	3.80	2	1.90	1.000	0.994	0.019
Model 7	11.13	6	1.855	0.995	0.981	0.019
Model 8	15.33	6	2.56	0.997	0.987	0.017

*CFI:* Comparative Fit Index, *TLI* :Tucker-Lewis Index, *RMSEA:* Root Mean Square Error of Approximation. Model 5 refers to the mediation model of RF_CGS on phonological awareness, Morphological awareness and RAN. Model 6 refers to the mediation model of RF_CGS on RANs. Model 7 refers to the mediation model of CR_CGS on phonological awareness, morphological awareness and RAN. Model 8 refers to the mediation model of CR_CGS on RANs

**Table 4:** Specific indirect effects of RANs, phonological awareness and morphological awareness from CGS to reading fluency. (Standardized βs and SEs are reported)

	β	SE	95% CI*
Model 5			
Total indirect effect	0.030	0.013	**[0.004, 0.055]**
RF_CGS → RAN → Reading fluency	0.025	0.011	**[0.001, 0.017]**
RF_CGS → Phonological awareness → Reading fluency	0.001	0.002	[−0.001, 0.002]
RF_CGS → Morphological awareness → Reading fluency	0.003	0.003	[−0.001, 0.003]
Model 6			
Total indirect effect	0.030	0.013	**[0.004, 0.056]**
RF_CGS → Digit RAN → Reading fluency	0.021	0.010	**[0.001, 0.041]**
RF_CGS → Dice RAN → Reading fluency	0.001	0.002	[−0.001, 0.006]
RF_CGS → Picture RAN → Reading fluency	0.004	0.003	[−0.001, 0.012]
RF_CGS → Color RAN → Reading fluency	0.003	0.002	[0.000, 0.008]

^*^Significant coefficients are reported in bold

We further analyzed four RAN tasks as parallel mediating variables. The results showed that the mediating effect of digit RAN (*p* = 0.04, *95% CI* [0.001, 0.041]) and total indirect effect (*p* = 0.03, *95% CI* [0.004, 0.056]) were significant in reading fluency (Model 6 in Table 4, Fig. 4B). Model 6 has a good model fit index: the CFI (comparative fit index) = 1.000 > 0.90; the TLI (Tucker‒Lewis index) = 0.994 > 0.90; the RMSEA (root mean square error of approximation) = 0.019 < 0.80; and χ^2^/*df* = 1.90 < 3 (Table 3). According to the β values, the specific indirect effect pathways were positive. The result suggested that the higher the RF_CGS, the longer the RAN time, and correspondingly, the longer the reading fluency time.

The mediation model from CR_CGS to character recognition showed that there were also significant mediating effects of RAN. Though the total indirect effect is not significant (*p* = 0.12, *95% CI* [-0.034, 0.003]), the mediating effect of RAN (average Z score of the four RAN tasks) was significant (Model 7 in Additional file 1: Table S5, Figure S1). In the separate RANs mediation model, the mediating effects of digit RAN (*p* = 0.03, *95% CI* [-0.019, -0.002]) and picture RAN were significant (*p* = 0.01, *95% CI* [0.004, 0.019]) (Model 8 in Additional file 1: Table S5, Figure S2).

## Discussion

The present study examined the cumulative effect of *KIAA0319* on reading skills by a moderation effect of parental educational level and a mediation effect of reading-related linguistic skills. The interaction between individual SNPs in *KIAA0319* and the parental education level was not significant. However, the interaction between the CGS of *KIAA0319* and the parental education level on reading fluency was significant, suggesting that *KIAA0319* may affect children's reading abilities through multiple minor effects. We also found that the CGS of *KIAA0319* can affect children's reading abilities through rapid automatized naming, mainly by digit rapid automatized naming. Additionally, we built four moderated mediation models according to the significant mediator variables and found that these models did not fit well (Additional file 1: Table S6 and Figure S3). These results suggested that *KIAA0319* influence behavioral phenotypes either through mediation model or moderation model independently.

The present study is the first to assess the cumulative effect of candidate gene *KIAA0319* on reading ability in Chinese children using the cumulative genetic risk score. As already noted, in most G × E work, one polymorphism is studied at a time. The CGS model accounts for more variance than individual SNPs, which, to a certain extent, reduced the problem of repeated analysis of individual polymorphisms [63]. In present study, the cumulative effect of *KIAA0319* only accounted for 0.1% variance and G × E effect accounted for 0.3% variance in reading fluency. The pathway from gene to reading behavior is far away and might be through complex and comprehensive mediation processes, it is reasonable that the cumulative effect of *KIAA0319* is very weak. This is indeed the reason that we should investigate the mediation phenotypes that could explain the relations between genes and behaviors [58].

In terms of G × E, individuals with a low CGS were better at reading fluency in a positive environment than individuals with a high CGS. These results suggested that the more risk alleles an individual carries, the worse their performance, and *KIAA0319* is theoretically a vulnerability gene. The G × E effect on reading fluency was found to be consistent with the differential susceptibility model by competing model analysis. This result indicated that “vulnerability genes” can be appropriately described as “plastic genes” because they make individuals more susceptible to environmental influences and thus exhibit better or worse behavior [23, 26]. In this study, the negative effects of *KIAA0319* on reading ability were verified for the first time through reparameterized regression models. In other words, the more risk alleles an individual carries, the more vulnerable they are, potentially causing irreversible harm, and the environment has little effect on the individual. Conversely, individuals carrying fewer vulnerability alleles are more susceptible to environmental influences and thus perform well.

To some extent, the finding is also consistent with the vantage sensitivity model due to the small value of the cross point. The notion is that salutary environments can moderate the influence of genetic variations on behaviors [25, 64] but not adverse environments [65]. Although there have been some findings of vantage sensitivity models in other domains, the study of cognitive ability has shown that the cumulative genetic scores of the *COMT* and *DRD2* genes and the effect of father authoritarianism on creativity are consistent with the vantage sensitivity model [66], the evidence is lacking in the field of reading. Therefore, the results of the vantage sensitivity model need to be further validated.

Finally, our study also supports the mediating role of digit RAN between *KIAA0319* and reading abilities [47, 67]. For the first time, we found that the cumulative effect of *KIAA0319* can affect Chinese word reading fluency and character recognition through RAN. The correlations between the CGS and RAN were consistent with previous results that *KIAA0319* can affect RAN [12, 52]. Although previous studies have also consistently reported RAN is an important predictor for Chinese reading accuracy [68] and reading fluency [34, 69] as well as for reading abilities in various orthographies [70–72], no study has examined whether RAN can mediate a gene-reading association. The current research provided the first hand evidence for RAN as a possible endophenotype between gene and behavioral phenotype. Our data also suggest that digit RAN might be the best RAN endophenotype among all RAN tasks. This is consistent with previous behavioral studies, in which digit RAN has been used more widely than other RAN tasks in predicting reading abilities and dyslexia [55, 57, 73]. However, it should be noted that we also found picture RAN can mediate *KIAA0319* and reading accuracy, but not for reading fluency. This suggests that other than digit RAN, sometimes picture RAN might also be able to serve as a surrogate endophenotype.

Previous studies have indicated that *KIAA0319* was mainly expressed in the cerebral cortex, amygdala and cerebellum [74–76], suggested the alternative level of *KIAA0319* could be the cause of neuronal migration abnormalities that might lead to the development of dyslexia. Furthermore, Jamadar et al. found a significant association between KIAA0139 and cerebellar gray matter volume in dyslexic patients [77]. Rapid automatized naming as an ability to retrieve familiar phonological information automatically is a reliable indicator of reading skills [67]. Cerebellum theory of dyslexia [78] provides a theoretical framework which indicates that cerebellum might be a key to automatic decoding and processing of words, which affects the accuracy and fluency of word reading [79]. Recently, our GWAS study discovered that *EVC* expression in the cerebellum affected reading fluency, further supporting the cerebellar theory [80]. Thus, it is reasonable to speculate that the cumulative effect might affect the expression of *KIAA0319* in the cerebellum, and in turn, impair the automated processing and reading speed.

Alternatively, *KIAA0319* has also been found to associate with the rapid auditory processing deficit of dyslexia [3, 81]. The expression of *KIAA0319* has been found to influence the temporal lobe [74, 81, 82], whose biological signals may have an impact on the neuronal temporal coding, and in turn, impact the auditory processing. Indeed, the rapid auditory processing deficit theory suggests that the individuals who are impaired in reading might be due to poor hearing for short and rapidly changing sounds [83]. Phoneme processing problems might result from imprecise acoustic input encoding [84]. Hence, phonological impairment of dyslexia is actually caused by rapid auditory processing deficit [83]. Therefore, an alternative explanation for the mediation effect between *KIAA0319*, RAN, and reading might be that *KIAA0319* have an impact on the expression in temporal lobe and auditory processing, then further affect RAN and reading.

There were several limitations in the current study. First, contrary to our findings, previous studies did not provide sufficient evidence for RAN as an EP [43, 85], whereas in our study, RAN was found to mediate gene-phenotype associations. This might be due to different measurement methods of endophenotypes, different languages and fewer selected SNPs in these studies. Second, we used β values derived from the phenotype of reading abilities, so the individual variant and cumulative genetic scores encoded do not provide an accurate estimate of the effect on RAN. This independent cumulative effect might affect the mediation effect of RAN, which means that, coincident with models of multiple deficits, the influence of genetic variation in reading ability through RAN, especially for rapid automatized naming tasks of color and dice is limited [1, 86]. Third, we did not find significant mediating effects of phonological awareness and morphological awareness. In this study we only used phoneme deletion and morphological production to test phonological awareness and morphological awareness. Future studies might be valuable to use other tasks (e.g., spoonerism, morphological judgment) to further investigate whether phonological awareness and morphological awareness can mediate gene and reading as endophenotypes. Finally, the SNPs we selected did not show significant interaction effects. A possible reason is the insufficient statistical power, since the number of samples verifying the interaction was more than the number of samples for which the effect was found alone [87]. The significant results of cumulative gene score might be the increased power of the cumulative effect, which should be replicated in an independent cohort to verify the interaction effects.

## Conclusion

*KIAA0139*, a candidate gene for reading ability, is a risk factor of reading disability, not a protective factor. The more risk alleles a person carries, the worse their reading fluency is. The finding that *KIAA0319* impacts reading fluency by interacting with parental education level suggests that environmental variables can modulate the effects of *KIAA0319* on children’s reading behaviors. Individuals with a low CGS of *KIAA0319* were better at reading fluency in a positive environment (higher parental educational level) than individuals with a high CGS. In addition, the impact of the genetic cumulative effect of *KIAA0319* on reading abilities can be mediated by cognitive intermediate phenotypes of rapid automatized naming. These findings provide evidence that *KIAA0319* is a risk vulnerability gene that interacts with environmental factor to impact reading abilities and demonstrate the reliability of RAN as an endophenotype between gene and reading associations.

## Supplementary Information

**Additional file 1: Table S1.** Characteristics of the single-nucleotide polymorphismsof KIAA0319. **Table S2. **Correlation matrix of cumulative genetic scores, cognitive skills, reading fluency, character recognition. **Table S3. **Results for standard parameterization models for 13 SNPs on reading fluency. **Table S4. **Results for standard parameterization models for 13 SNPs and CR_CGS on character recognition. **Table S5. **Specific indirect effects of RANs, phonological awareness and morphological awareness from CGS to character recognition in modified models. **Table S6.** Fitting index of cumulative genetic score of Reading fluency on moderated mediation model. **Figure S1. **Significant specific indirect of the RAN effect from CR_CGS to character recognition after controlling for sex and age. **Figure S2.** Significant specific indirect effects of the digit RAN and picture RAN from CR_CGS to character recognition after controlling for sex and age. The model was adjusted for the correlations between different RAN tasks. **Figure S3**. The moderated-mediation model of Parental Education and RAN.

## Data Availability

Codes used in this study are available from the authors upon request.
