# Supplementary material for: Cumulative genetic score of KIAA0319 affects reading ability in Chinese children: moderation by parental education and mediation by rapid automatized naming
Source: Behav Brain Funct. 2023 Jun 1;19:10. doi: 10.1186/s12993-023-00212-z (PMC10234066; doi:10.1186/s12993-023-00212-z)
Supplement: Supplementary file 1 — Additional file 1: Table S1. Characteristics of the single-nucleotide polymorphismsof KIAA0319. Table S2. Correlation matrix of cumulative genetic scores, cognitive skills, reading fluency, character recognition. Table S3. Results for standard parameterization models for 13 SNPs on reading fluency. Table S4. Results for standard parameterization models for 13 SNPs and CR_CGS on character recognition. Table S5. Specific indirect effects of RANs, phonological awareness and morphological awareness from CGS to character recognition in modified models. Table S6. Fitting index of cumulative genetic score of Reading fluency on moderated mediation model. Figure S1. Significant specific indirect of the RAN effect from CR_CGS to character recognition after controlling for sex and age. Figure S2. Significant specific indirect effects of the digit RAN and picture RAN from CR_CGS to character recognition after controlling for sex and age. The model was adjusted for the correlations between different RAN tasks. Figure S3. The moderated-mediation model of Parental Education and RAN. [file 12993_2023_212_MOESM1_ESM.docx]

**Table S1.** Characteristics of the single-nucleotide polymorphisms (SNPs) of KIAA0319.

| SNP | Position | A1 | A2 | HWE *p* | Typed/Imputation | β_1_ | *p_1_* | β_2_ | *p_2_* |
| --- | --- | --- | --- | --- | --- | --- | --- | --- | --- |
| rs2038137 | 6:24645943 | T | G | .160 | I | -1.55 | .04 | .77 | .55 |
| rs2038139 | 6:24645420 | C | A | .158 | I | -1.41 | .06 | .56 | .66 |
| rs6935076 | 6:24644322 | T | C | .228 | T | .55 | .83 | 1.82 | .11 |
| rs16889556 | 6:24641605 | T | C | .156 | I | .68 | .38 | 1.22 | .35 |
| rs9366577 | 6:24641328 | C | T | .052 | T | .21 | .17 | 2.53 | .24 |
| rs761100 | 6:24632642 | A | C | .290 | T | -1.66 | .02 | .59 | .64 |
| rs2179515 | 6:24628203 | T | C | .571 | I | -1.50 | .04 | .64 | .62 |
| rs16889506 | 6:24595853 | C | T | .551 | I | 1.10 | .14 | -2.04 | .11 |
| rs4504469 | 6:24588884 | T | C | 162 | T | -1.48 | .04 | .76 | .54 |
| rs807507 | 6:24579867 | G | C | .100 | I | -.28 | .65 | -.23 | .83 |
| rs2760157 | 6:24578272 | A | G | .493 | I | .11 | .83 | .49 | .60 |
| rs12193738 | 6:24568393 | C | T | .042 | I | -.26 | .69 | -.53 | .62 |
| rs699463 | 6:24544903 | A | G | .422 | T | -.06 | .93 | -.34 | .81 |

Note: HWE = Hardy-Weinberg equilibrium; *p* (HWE) >0.05 means the population genetic balance and the sample is representative; β_1_ and *p_1_* refer to Character Recognition’s beta value; β_2_ and *p_2_* refer to Reading Fluency’s beta value; T/I:Genotyped/Imputation.

**Table S2.** Correlation matrix of cumulative genetic scores, cognitive skills, reading fluency, character recognition.

|  |  | CR_CGS | RF_CGS | RF | CR | PA | MA | R1 | R2 | R3 | R4 | R-mean | SEX | AGE | PE |
| --- | --- | --- | --- | --- | --- | --- | --- | --- | --- | --- | --- | --- | --- | --- | --- |
| CR_CGS | Pearson | 1 |  |  |  |  |  |  |  |  |  |  |  |  |  |
|  | Sig |  |  |  |  |  |  |  |  |  |  |  |  |  |  |
|  | FDR |  |  |  |  |  |  |  |  |  |  |  |  |  |  |
| RF_CGS | Pearson | **-.251^**^** | 1 |  |  |  |  |  |  |  |  |  |  |  |  |
|  | Sig | P<.001 |  |  |  |  |  |  |  |  |  |  |  |  |  |
|  | FDR | P<.001 |  |  |  |  |  |  |  |  |  |  |  |  |  |
| RF | Pearson | .006 | .040 | 1 |  |  |  |  |  |  |  |  |  |  |  |
|  | Sig | .777 | .056 |  |  |  |  |  |  |  |  |  |  |  |  |
|  | FDR | .794 | .090 |  |  |  |  |  |  |  |  |  |  |  |  |
| CR | Pearson | .029 | -.039 | **-.475^**^** | 1 |  |  |  |  |  |  |  |  |  |  |
|  | Sig | .174 | .065 | P<.001 |  |  |  |  |  |  |  |  |  |  |  |
|  | FDR | .232 | .095 | P<.001 |  |  |  |  |  |  |  |  |  |  |  |
| PA | Pearson | .011 | -.011 | **-.262^**^** | **.307^**^** | 1 |  |  |  |  |  |  |  |  |  |
|  | Sig | .596 | .595 | P<.001 | P<.001 |  |  |  |  |  |  |  |  |  |  |
|  | FDR | .631 | .631 | P<.001 | P<.001 |  |  |  |  |  |  |  |  |  |  |
| MA | Pearson | -.009 | -.029 | **-.273^**^** | **.310^**^** | **.368^**^** | 1 |  |  |  |  |  |  |  |  |
|  | Sig | .670 | .188 | P<.001 | P<.001 | P<.001 |  |  |  |  |  |  |  |  |  |
|  | FDR | .692 | .248 | P<.001 | P<.001 | P<.001 |  |  |  |  |  |  |  |  |  |
| R1 | Pearson | .041 | **.052^*^** | **.559^**^** | **-.296^**^** | **-.206^**^** | **-.175^**^** | 1 |  |  |  |  |  |  |  |
|  | Sig | .061 | .017 | P<.001 | P<.001 | P<.001 | P<.001 |  |  |  |  |  |  |  |  |
|  | FDR | .095 | .030 | P<.001 | P<.001 | P<.001 | P<.001 |  |  |  |  |  |  |  |  |
| R2 | Pearson | .041 | .036 | **.393^**^** | **-.256^**^** | **-.189^**^** | **-.165^**^** | **.539^**^** | 1 |  |  |  |  |  |  |
|  | Sig | .062 | .108 | P<.001 | P<.001 | P<.001 | P<.001 | P<.001 |  |  |  |  |  |  |  |
|  | FDR | .095 | .154 | P<.001 | P<.001 | P<.001 | P<.001 | P<.001 |  |  |  |  |  |  |  |
| R3 | Pearson | .041 | .035 | **.399^**^** | **-.264^**^** | **-.169^**^** | **-.187^**^** | **.452^**^** | **.557^**^** | 1 |  |  |  |  |  |
|  | Sig | .065 | .116 | P<.001 | P<.001 | P<.001 | P<.001 | P<.001 | P<.001 |  |  |  |  |  |  |
|  | FDR | .095 | .163 | P<.001 | P<.001 | P<.001 | P<.001 | P<.001 | P<.001 |  |  |  |  |  |  |
| R4 | Pearson | **.053^*^** | **.056^*^** | **.370^**^** | **-.238^**^** | **-.192^**^** | **-.185^**^** | **.447^**^** | **.540^**^** | **.623^**^** | 1 |  |  |  |  |
|  | Sig | .019 | .012 | P<.001 | P<.001 | P<.001 | P<.001 | P<.001 | P<.001 | P<.001 |  |  |  |  |  |
|  | FDR | .032 | .023 | P<.001 | P<.001 | P<.001 | P<.001 | P<.001 | P<.001 | P<.001 |  |  |  |  |  |
| R-mean | Pearson | **.061^**^** | **.055^*^** | **.541^**^** | **-.325^**^** | **-.242^**^** | **-.226^**^** | **.762^**^** | **.823^**^** | **.820^**^** | **.814^**^** | 1 |  |  |  |
|  | Sig | .007 | .016 | P<.001 | P<.001 | P<.001 | P<.001 | P<.001 | P<.001 | P<.001 | P<.001 |  |  |  |  |
|  | FDR | .001 | .028 | P<.001 | P<.001 | P<.001 | P<.001 | P<.001 | P<.001 | P<.001 | P<.001 |  |  |  |  |
| SEX | Pearson | -.017 | .012 | .005 | .029 | **.054^*^** | -.015 | **.050^*^** | .014 | **-.086^**^** | -.077^**^ | -.037 | 1 |  |  |
|  | Sig | .400 | .559 | .795 | .164 | .011 | .481 | .022 | .537 | P<.001 | .001 | .101 |  |  |  |
|  | FDR | .467 | .613 | .804 | .223 | .020 | .548 | .037 | .596 | P<.001 | P<.001 | .146 |  |  |  |
| AGE | Pearson | -.024 | -.038 | .032 | .021 | **-.080^**^** | **-.094^**^** | -.023 | **-.075^**^** | **-.050^*^** | **-.048^*^** | **-.054^*^** | -.009 | 1 |  |
|  | Sig | .236 | .062 | .130 | .317 | P<.001 | P<.001 | .285 | .001 | .025 | .033 | .018 | .669 |  |  |
|  | FDR | .298 | .095 | .180 | .374 | P<.001 | P<.001 | .349 | .001 | .040 | .054 | .032 | .692 |  |  |
| PE | Pearson | .003 | .032 | **-.139^**^** | .030 | **.146^**^** | **.200^**^** | -.030 | .019 | -.017 | -.028 | -.015 | -.026 | **-.387^**^** | 1 |
|  | Sig | .898 | .204 | P<.001 | .225 | P<.001 | P<.001 | .244 | .468 | .530 | .289 | .584 | .291 | P<.001 |  |
|  | FDR | .898 | .265 | P<.001 | .288 | P<.001 | P<.001 | .304 | .539 | .595 | .349 | .248 | .349 | P<.001 |  |

CR_CGS: the cumulative genetic socre of KIAA0319 on character recognition; RF_CGS: the cumulative genetic socre of KIAA0319 on reading fluency; RF: reading fluency; CR: character recognition; PA: phonological awareness; MA: morphological awareness; R1: digit rapid automatized naming; R2: dice rapid automatized naming; R3: picture rapid automatized naming; R4: color rapid automatized naming；R-mean: the mean Z-score of four RANs; PE: parental education. The bold indicates that the correlations are significant and reach FDR correction.

**Table S2.** Results for standard parameterization models for 13 SNPs on reading fluency.

|  | Gene(G) and environment(E) main effects: Model 1 | | | | | | | Main effects and G×E interaction: Model 2 | | | | | | | | |
| --- | --- | --- | --- | --- | --- | --- | --- | --- | --- | --- | --- | --- | --- | --- | --- | --- |
|  | *B_1_* | *p* | *B_2_* | *p* | *R^2^* | *F* | *df* | *B_1_* | *p* | *B_2_* | *p* | *B_3_* | *p* | *R^2^* | *F* | *df* |
| rs699463 | -5.25 | <.0001 | 1.58 | .344 | .083 | 36.04 | 4,1583 | -7.66 | .002 | -3.09 | .524 | 1.42 | .305 | .084 | 29.04 | 5,1582 |
| rs12193738 | -5.24 | <.0001 | .76 | .560 | .083 | 35.89 | 4,1583 | -5.90 | .001 | -.65 | .863 | .44 | .691 | .083 | 28.73 | 5,1582 |
| rs2760157 | -5.25 | <.0001 | 1.03 | .375 | .083 | 36.03 | 4,1583 | -5.43 | <.0001 | .34 | .917 | .21 | .823 | .083 | 28.81 | 5,1582 |
| rs807507 | -5.24 | <.0001 | -.38 | .768 | .083 | 35.82 | 4,1583 | -6.13 | .001 | -1.52 | .687 | .59 | .591 | .083 | 28.70 | 5,1582 |
| rs4504469 | -5.25 | <.0001 | .66 | .646 | .083 | 35.85 | 4,1583 | -5.59 | <.0001 | -2.51 | .555 | .95 | .428 | .083 | 28.80 | 5,1582 |
| rs16889506 | -5.26 | <.0001 | 2.25 | .142 | .084 | 36.38 | 4,1583 | -8.36 | <.0001 | -3.72 | .384 | 1.86 | .135 | .085 | 29.58 | 5,1582 |
| rs2179515 | -5.25 | <.0001 | -.02 | .989 | .083 | 35.80 | 4,1583 | -5.83 | <.0001 | -6.43 | .160 | 1.94 | .137 | .084 | 29.10 | 5,1582 |
| rs761100 | -5.24 | <.0001 | .21 | .885 | .083 | 35.80 | 4,1583 | -5.75 | <.0001 | -5.39 | .225 | 1.56 | .217 | .084 | 28.95 | 5,1582 |
| rs9366577 | -5.25 | <.0001 | 2.03 | .423 | .083 | 35.97 | 4,1583 | -5.17 | <.0001 | 4.25 | .551 | -.68 | .738 | .083 | 28.78 | 5,1582 |
| rs16889556 | -5.25 | <.0001 | 3.09 | .050 | .085 | 36.84 | 4,1583 | -5.67 | <.0001 | -1.42 | .762 | 1.37 | .305 | .086 | 29.69 | 5,1582 |
| rs6935076 | -5.26 | <.0001 | 3.16 | .021 | .086 | 37.24 | 4,1583 | -5.51 | <.0001 | 1.44 | .722 | .52 | .650 | .086 | 29.82 | 5,1582 |
| rs2038139 | -5.25 | <.0001 | -.15 | .924 | .083 | 35.80 | 4,1583 | -5.88 | <.0001 | -7.13 | .118 | 2.13 | 1.04 | .084 | 29.20 | 5,1582 |
| rs2038137 | -5.25 | <.0001 | .13 | .932 | .083 | 35.80 | 4,1583 | -5.84 | <.0001 | -6.27 | .169 | 1.95 | .136 | .084 | 29.10 | 5,1582 |

**Table S4.** Results for standard parameterization models for 13 SNPs and CR_CGS on character recognition.

|  | Gene(G) and environment(E) main effects: Model 1 | | | | | | | Main effects and G×E interaction: Model 2 | | | | | | | | |
| --- | --- | --- | --- | --- | --- | --- | --- | --- | --- | --- | --- | --- | --- | --- | --- | --- |
|  | *B_1_* | *p* | *B_2_* | *p* | *R^2^* | *F* | *df* | *B_1_* | *p* | *B_2_* | *p* | *B_3_* | *p* | *R^2^* | *F* | *df* |
| rs699463 | 2.11 | <.0001 | -.96 | .320 | .228 | 116.73 | 4,1584 | 1.92 | .178 | -1.33 | .636 | .11 | .890 | .228 | 93.33 | 5,1583 |
| rs12193738 | 2.11 | <.0001 | .46 | .541 | .227 | 116.53 | 4,1584 | 1.43 | .176 | -1.00 | .650 | .45 | .479 | .228 | 93.29 | 5,1583 |
| rs2760157 | 2.11 | <.0001 | -.30 | .643 | .227 | 116.48 | 4,1584 | 2.07 | .001 | -.43 | .820 | .40 | .941 | .227 | 93.12 | 5,1583 |
| rs807507 | 2.11 | <.0001 | .50 | .503 | .227 | 116.55 | 4,1584 | 1.52 | .149 | -.77 | .725 | .39 | .535 | .228 | 93.28 | 5,1583 |
| rs4504469 | 2.12 | <.0001 | 1.11 | .182 | .228 | 116.98 | 4,1584 | 1.70 | .164 | .27 | .911 | .25 | .718 | .228 | 93.56 | 5,1583 |
| rs16889506 | 2.11 | <.0001 | 1.29 | .147 | .228 | 117.09 | 4,1584 | 1.88 | <.0001 | -.96 | .700 | .70 | .333 | .229 | 93.85 | 5,1583 |
| rs2179515 | 2.11 | <.0001 | 1.23 | .162 | .228 | 117.04 | 4,1584 | 1.43 | .291 | -1.00 | .970 | .40 | .594 | .228 | 93.65 | 5,1583 |
| rs761100 | 2.11 | <.0001 | 1.29 | .130 | .228 | 117.15 | 4,1584 | 1.66 | .201 | .388 | .880 | .27 | .711 | .228 | 93.69 | 5,1583 |
| rs9366577 | 2.10 | <.0001 | .63 | .671 | .227 | 116.46 | 4,1584 | 1.99 | <.0001 | -2.48 | .547 | .94 | .419 | .228 | 93.28 | 5,1583 |
| rs16889556 | 2.10 | <.0001 | .78 | .394 | .228 | 116.64 | 4,1584 | 2.45 | <.0001 | 4.57 | .092 | -1.15 | .138 | .229 | 93.82 | 5,1583 |
| rs6935076 | 2.10 | <.0001 | .79 | .325 | .228 | 116.71 | 4,1584 | 2.35 | <.0001 | 2.57 | .271 | -.54 | .416 | .228 | 93.49 | 5,1583 |
| rs2038139 | 2.11 | <.0001 | 1.10 | .213 | .228 | 116.91 | 4,1584 | 1.02 | .451 | -1.00 | .705 | .64 | .398 | .228 | 93.65 | 5,1583 |
| rs2038137 | 2.11 | <.0001 | 1.27 | .151 | .228 | 117.07 | 4,1584 | 1.43 | .288 | -.06 | .983 | .40 | .595 | .228 | 93.67 | 5,1583 |
| CR_CGS | 2.09 | <.0001 | .17 | .147 | .228 | 116.50 | 4,1584 | 1.30 | .426 | .00 | .994 | 0.05 | .620 | .228 | 93.24 | 5,1583 |

**Table S5.** Specific indirect effects of RANs, phonological awareness and morphological awareness from CGS to character recognition in modified models.

|  | β | SE | 95%CI* |
| --- | --- | --- | --- |
| Model 7 |  |  |  |
| Total indirect effect | -.015 | .009 | [-.034, .003] |
| CR_CGS → RAN → Character recognition | -.014 | .006 | **[-.026, -.003]** |
| CR_CGS → Phonological awarenes→ Character recognition | .002 | .004 | [-.006, .009] |
| CR_CGS → Morphological awareness → Character recognition | -.003 | .004 | [-.011 .005] |
| Model8 |  |  |  |
| Total indirect effect | .004 | .008 | [-.013, .020] |
| CR_CGS → Digit RAN → Character recognition | -.010 | .004 | **[-.019, -.002]** |
| CR_CGS →Dice RAN → Character recognition | -.001 | .002 | [-.005, .002] |
| CR_CGS → Picture RAN → Character recognition | .010 | .004 | **[.004, .019]** |
| CR_CGS → Color RAN → Character recognition | .004 | .003 | [.000, .010] |

**Table S6.** Fitting index of cumulative genetic score of Reading fluency on moderated mediation model.

|  | χ2 | df | χ2/df | CFI | TLI | RMSEA |
| --- | --- | --- | --- | --- | --- | --- |
| Model S1 | 324.81 | 10 | 32.48 | .625 | .212 | .147 |
| Model S2 | 324.92 | 11 | 29.54 | .626 | .286 | .140 |
| Model S3 | 834.25 | 12 | 69.50 | .420 | -.160 | .221 |
| Model S4 | 834.26 | 13 | 64.17 | .421 | -.069 | .213 |


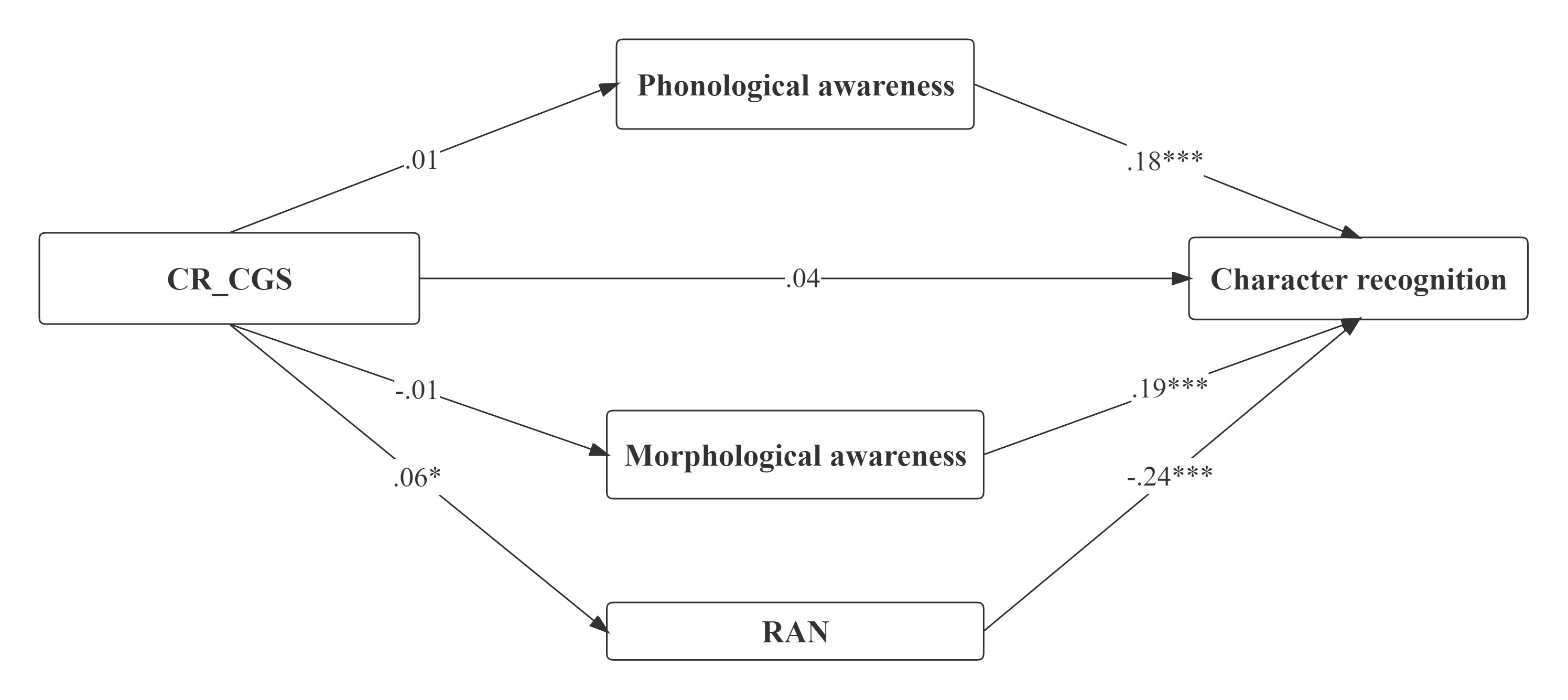


**Figure S1.** Significant specific indirect of the RAN effect from CR_CGS to character recognition after controlling for sex and age (standardized estimates of the path coefficients are depicted in Model 7).


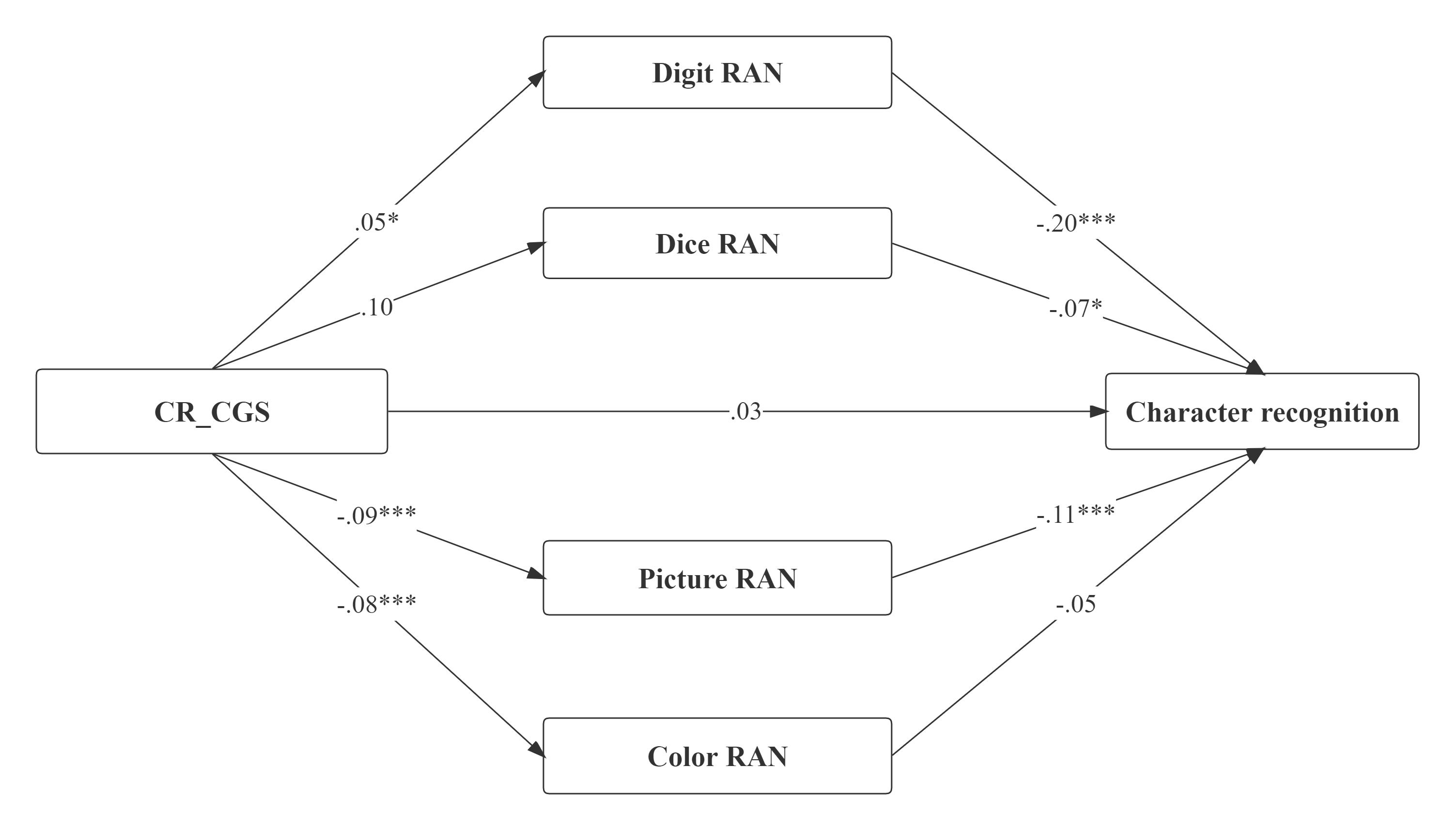


**Figure S2.** Significant specific indirect effects of the digit RAN and picture RAN from CR_CGS to character recognition after controlling for sex and age (standardized estimates of the path coefficients are depicted in Model 8). The model was adjusted for the correlations between different RAN tasks (some path coefficients have been omitted for brevity).


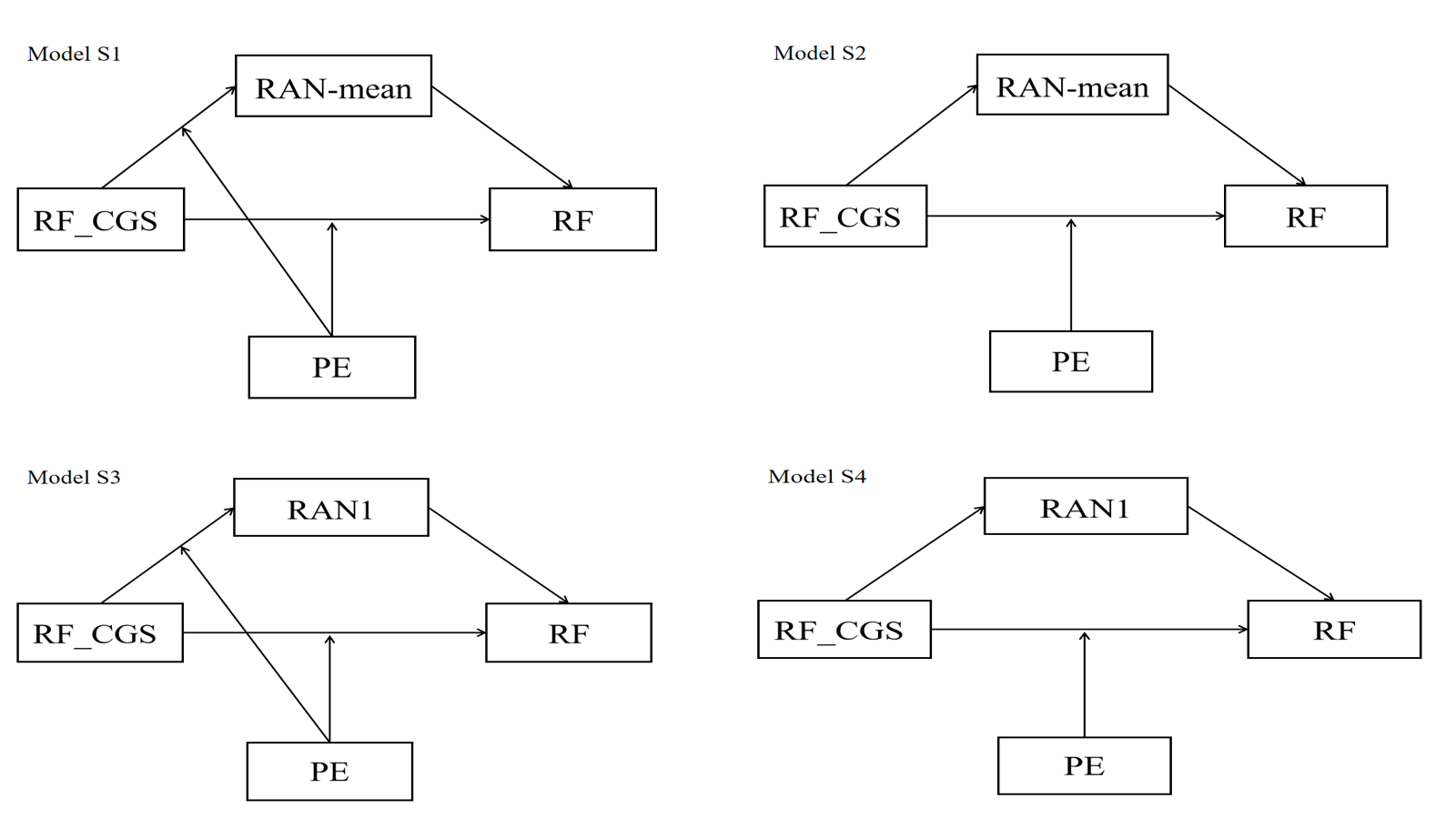


**Figure S3**. The moderated-mediation model of Parental Education and RAN.
